# Mini Review: Transport of Hydrophobic Polymers Into the Plant Apoplast

**DOI:** 10.3389/fpls.2020.590990

**Published:** 2021-01-07

**Authors:** Anzhou Xin, Klaus Herburger

**Affiliations:** ^1^The Edinburgh Cell Wall Group, Institute of Molecular Plant Sciences, School of Biological Sciences, The University of Edinburgh, Edinburgh, United Kingdom; ^2^Section for Plant Glycobiology, Department of Plant and Environmental Sciences, University of Copenhagen, Copenhagen, Denmark

**Keywords:** lignin, suberin, cutin, cutan, transmembrane export, ABC transporters, LTPs, cutinsome

## Abstract

The plant apoplast contains the four hydrophobic polymer, lignin, suberin, cutin, and cutan, that are crucial for stress resistance, controlling solute diffusion, and strengthening the cell wall. Some of these polymers are widely used in industry and daily life products, such as all wood-containing goods (lignin) and wine cork (suberin). Despite the importance of these polymers, several aspects of their formation remain unknown. This mini review highlights technical bottlenecks in the current research and summarizes recent insights into the precursor transmembrane transport, an essential step in the polymer formation. We also briefly discuss how some of the remaining knowledge gaps can be closed and how a better understanding of these biopolymers will benefit other research fields.

## Introduction

**FIGURE 1:**
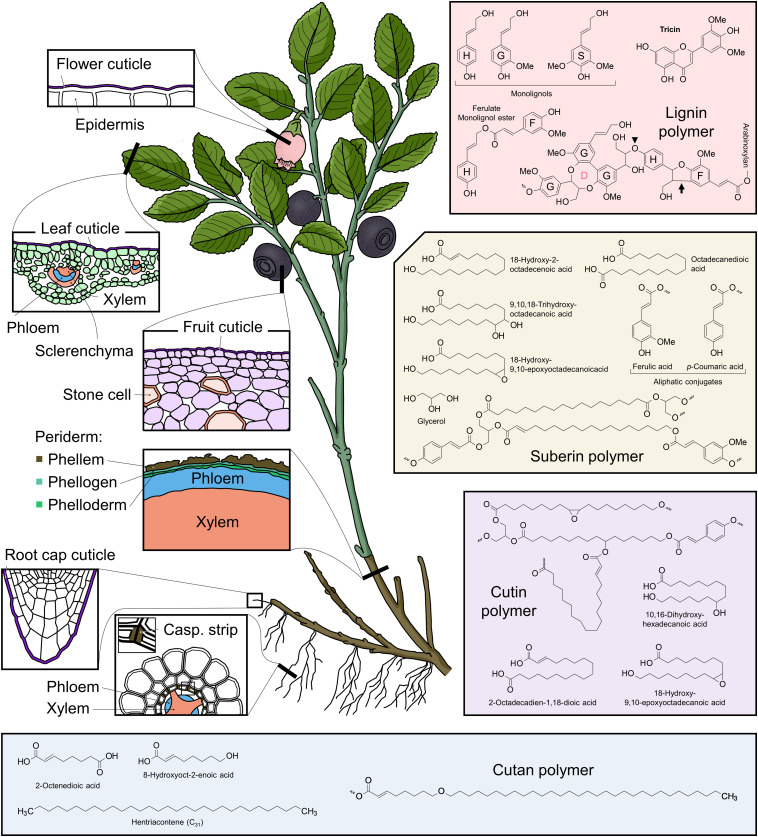
Overview of the localizations of lignin, suberin, cutin, and cutan; their major precursors; and hypothetical polymer structures. Major organ- and tissue-specific localizations of the four hydrophobic biopolymers are as follows: lignin in vascular bundles of shoots and roots, in structural tissues, such as leaf sclerenchyma, and in idioblasts, such as fruit stone cells (Kumar et al., 2016); suberin in stem periderm and root endodermis (Casparian strip; Franke and Schreiber, 2007); cutin in the epidermal cuticle of leaves (Bourgault et al., 2020) and flowers (“nanobridges”; Li-Beisson et al., 2016) and in the root cap (Berhin et al., 2019); and cutan in the leaf and fruit cuticle of some species, such as *Vaccinium myrtillus* (Boom et al., 2005; Kallio et al., 2006). Examples of common constituents of the four biopolymers are as follows: lignin: three monolignols (H, G, and S), ferulate monolignol ester (similar to coumarate ester, not shown), and tricin represent lignin building blocks. A lignin polymer with ethers and C–C as the major bond types is represented here. The dibenzodioxocin (D) unit, an eight-membered ring, contains most of the 5–5 (C–C) bonds in lignin. The β-O-4 ether bond is prevalent in lignin, e.g., linking G and H monolignols (arrowhead). The β-5 (C–C) link is illustrated by H monolignol and ferulate (F) bonding (arrow) (Ralph et al., 2019). Lignin building blocks are polymerized in the apoplast by oxidative coupling reactions mediated by peroxidases and laccases (Tobimatsu and Schuetz, 2019). Additional hydroxy groups (–OH) from water are introduced to the αC of the side chains through quinone methide re-aromatization after the coupling reaction (Tobimatsu and Schuetz, 2019). The F, which is oxidatively coupled to, e.g., H residues, can cross-link lignin to neighboring arabinoxylans in the cell wall through an ester bond (Ralph et al., 2019). Wiggle lines indicate unspecified ether bonds. Suberin: the predominant aliphatic-domain of suberin is represented by C_18_ fatty acids with mid-chain or ε-modifications (Franke et al., 2005; Pinto et al., 2009); ferulic acid and *p*-coumaric acid are the known aromatic constituents of suberin (Franke et al., 2005). Glycerol is a minor component (Franke and Schreiber, 2007) and might be ester-bonded to the fatty acids, potentially at both termini of α, ε dicarboxylic acids. The fatty acid chains attached to the same glycerol molecule could therefore form a local lamellar structure, contributing to the macromolecular structure (Graça and Santos, 2007). Aromatic acids are believed to ester bond to the hydroxy terminus of the fatty acids and glycerol molecules (Graça and Santos, 2007). Currently, there is no proposed role of monolignols and fatty alcohols (not shown) in suberin, but theoretically, they could behave as glycerol molecules (i.e., hydroxy groups ester bond with the carboxy groups of the fatty acids). An increasing number of studies suggest that GDSL family transacylases contribute to suberin polymerization (Lashbrooke et al., 2016; Ursache et al., 2020). Wiggle lines indicate unspecified ester bonds. Cutin: the dominant components are C_16_ and C_18_ hydroxy fatty acids; glycerol and hydroxycinnamic acids as minor components as in suberin. The macromolecular structure is not fully resolved yet, but glycerol is suggested to act as a scaffold for hydroxy fatty acids to form a lamellar structure as in suberin (Graça et al., 2002; Philippe et al., 2016), and dicarboxylic acids are proposed to cross-link the lamellas (Fich et al., 2016). Structural roles of ferulic acid and *p*-coumaric acid are unknown; here, they (exemplified by *p*-coumaric acid) are proposed to ester bond to hydroxy fatty acids as in suberin. Cutin polymerization through ester bonds is catalyzed by the GDSL family transacylase CUS1 (Yeats et al., 2012). Wiggle lines indicate unspecified ester bonds. Cutan: the non-polar domain is represented by C_31_ alkane and alkene (Boom et al., 2005); the hydroxy domain by C_8_ fatty acids (Villena et al., 1999). The proposed representative polymer structure contains possible ester bond (left wiggle) (Guzmán-Delgado et al., 2016), ether bond (middle C–O–C), and C–C bond (right wiggle). There is no information of how cutan is polymerized.

Plants secrete hydrophobic compounds into the apoplast, where they contribute substantially to the plant’s structural strength and resistance against environmental stresses (Kumar et al., 2016; Li-Beisson et al., 2016; Figure 1). Both lignin and suberin depositions are modifications of the secondary cell walls of land plants (Kumar et al., 2016; Figure 1), corroborating their function in rigidifying tissues predominated by dead cells (xylem, periderm; Figure 1). Lignin might exhibit similar functions in certain red and streptophyte green algae too (Martone et al., 2009; Sørensen et al., 2011). In contrast to lignin and suberin, cutin forms an “expandable” matrix on the plant surface as its deposition starts at early developmental stages (Casado and Heredia, 2001) and peaks during rapid growth phases (Yeats et al., 2012). Cutin is a major component of the leaf, shoot, and fruit epidermis (Figure 1) and important for limiting water loss and pathogen invasion (Philippe et al., 2020). Furthermore, it occurs in root caps as found recently (Berhin et al., 2019; Figure 1). Knowledge on cutan is very scarce, potentially owing to its limited phylogenetic spread and restriction to drought-adapted crassulacean acid metabolism (CAM) plants (Boom et al., 2005; Kallio et al., 2006; Figure 1).

An evolutionary interlink between lignin, suberin, and cutin was raised recently, because their precursor biosynthesis might share a set of enzymes in the moss *Physcomitrella patens* (Renault et al., 2017). Moreover, the aliphatic precursors of both suberin and cutin might be synthesized in the endoplasmic reticulum (ER), whereas their aromatic precursors, together with lignin precursors, are synthesized in the cytosol (Niklas et al., 2017; Philippe et al., 2020). To form lignin, suberin, cutin, and cutan polymers, their precursors must pass the plasma membrane and parts of the cell wall. In this mini review, we will discuss and compare the latest findings in precursor transmembrane transport mechanisms. Understanding this part of the polymer trafficking pipeline is crucial to better understand the polymer formation in the apoplast.

## Current Knowledge and Open Questions in Transmembrane Trafficking

### Lignin

Lignin protects plants from environmental stresses and allows them to construct a sophisticated solute transport system that withstands highly negative water potentials and provides structural strength (Liu et al., 2018; Figure 1). It consists of complex ether- and C–C-linked hydroxycinnamyl alcohols (i.e., monolignols that form the corresponding H, G, and S units in lignin; Kumar et al., 2016; Figure 1). Furthermore, glucosylated monolignols (e.g., coniferin) may serve as intermediates for lignin polymerization in some plants, especially gymnosperms (Ibrahim, 1977). Additional lignin building units, such as monolignol–ferulate ester (Karlen et al., 2016) and the flavone tricin (del Río et al., 2012), were confirmed recently (Figure 1).

**FIGURE 2:**
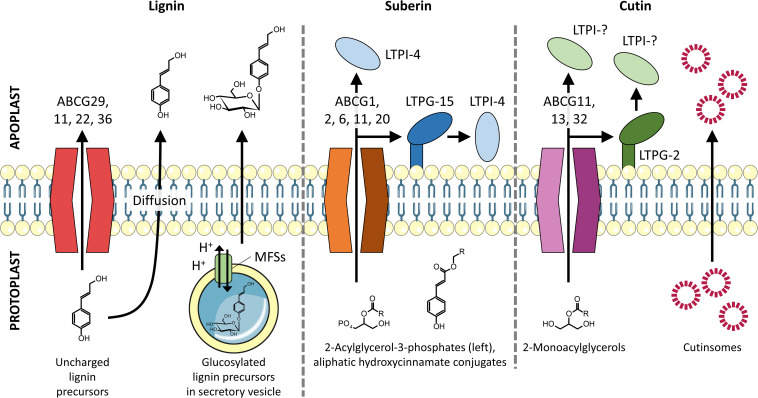
Transmembrane export of lignin, suberin, and cutin precursors into the apoplast. Lignin monomers are proposed to be exported by full-size ABC transporters, vesicle-mediated exocytosis, and passive diffusion. Suberin precursors are found to be exported by half-size ABC transporters with assistance from LTPs. Cutin precursors are exported by both full- and half-size ABC transporters and are proposed to pass through the hydrophilic cell wall by binding to LTPs and/or self-aggregate to form cutinsomes, which have a hydrophilic shell (Domínguez et al., 2010).

Four models for lignin precursor transport into the apoplast have been proposed (Liu, 2012; Perkins et al., 2019; Figure 2): (1) active transport *via* ATP-driven porters [e.g., ATP-binding cassette (ABC) transporters], (2) transport *via* cargo vesicles, (3) passive diffusion through the membrane, and/or (4) channel-facilitated transport. Considering that different species and even organs can contain a variety of lignin precursors (Dima et al., 2015; Vanholme et al., 2019), it is likely that non-specific and/or more than one transporting mechanism is required.

The first model has been supported in *Arabidopsis* leaf- and *Populus* root-derived plasma membrane vesicles that showed an ATP-dependent uptake of monolignols (Miao and Liu, 2010). This study is convincing because the vesicles retained a native lipid bilayer environment and ABC transporter inhibitors reduced the uptake. However, omitting ATP still resulted in some uptake of lignin precursors, indicating the presence of additional export mechanisms. The significance of ABC transporters for lignin export is further supported by identifying the monolignol ABC transporter AtABCG29 in *Arabidopsis* roots and stems; loss of function mutants showed a strongly reduced root length and lignin content (Alejandro et al., 2012). The root growth was significantly reduced due to the intracellular accumulation of H monolignol. In contrast, stems were not affected, supporting the idea that different organs rely on different transporters and/or mechanisms for trafficking lignin precursors. A recent study found that *Arabidopsis* cell cultures undergoing differentiation into lignin-rich tracheary elements co-upregulated the transporters AtABCG11, 22, and 36 with AtABCG29, indicating their roles in lignin precursor transport (Takeuchi et al., 2018). Loss-of-function mutants of AtABCG11, 22, and 36 will be required to demonstrate their physiological significance, and transport assays will help to assess their biochemical activities. Thus, until now, AtABCG29 is the only confirmed transporter for lignin precursors. This relatively slow progress in elucidating active lignin transport is surprising as omic tools (e.g., comparative transcriptomics) are readily available to screen for transporter candidate genes. A potential challenge is the redundancy of ABC transporters (e.g., ∼130 genes in *Arabidopsis*; Hwang et al., 2016). This bears the risk that single-knockout/down mutants do not show phenotypes, whereas generating multi-gene mutants could be time consuming. To bypass this bottleneck, ancestral unicellular algae could serve as model systems as they contain much fewer ABC transporters (e.g., <70 in *Chlamydomonas*; Hwang et al., 2016). The capability of algal ABC transporters to use lignin precursors as cargos could inform the search for homologs in lignified vascular plants.

The second model (exocytosis *via* vesicles) is somewhat counterintuitive to the widely accepted concept that lignin precursors are synthesized in the cytoplasm (Kumar et al., 2016). Interestingly, two recent studies suggested that glucosylated monolignols are loaded into secretory vesicles by an ATP-dependent proton gradient (V-ATPase) before being secreted, whereas monolignols are not (Tsuyama et al., 2013, 2019). Some efforts were made to identify the responsible porters. Väisänen et al. (2020) suggested major facilitator superfamily (MFS) transporters – proton gradient-dependent porters that specifically transported glucosylated monolignols through the tonoplast in Norway spruce cells that underwent differentiation into vascular cells. These findings are intriguing and corroborate the vacuolar storage of glucosylated monolignols (Perkins et al., 2019). However, further questions have to be addressed to underpin the idea of vesicle-mediated lignin secretion: (1) do glucosylated monolignols and the proposed transport machinery indeed co-localize in secretory vesicles *in planta*, (2) to what extent does vesicle-mediated lignin precursor secretion contribute to total precursor secretion, and (3) what is the physiological significance of utilizing both cytoplasmic- and vesicle-mediated transport routes for precursor secretion? A proton porter may not be the only mechanism for sequestering glucosylated monolignols. Miao and Liu (2010) showed that the ABC transporter inhibitor vanadate strongly reduced the uptake of glucosylated monolignols by vacuolar vesicles in *Arabidopsis* root cells. However, this drug did not affect the same transport mechanisms in differentiating xylem cells of cypress and spruce (Tsuyama et al., 2013, 2019). These discrepancies suggest that angiosperms rely on ABC transporters to sequester glucosylated monolignols, whereas gymnosperms use alternatives, such as MFSs in vesicle routes. Strategies to solve these puzzles might take advantage of fluorescently tagged candidate lignin transporters in combination with fluorescent or click chemistry-ready monolignols (Tobimatsu et al., 2013; Lion et al., 2017). This would allow for following the lignin precursor trafficking through the protoplast and into the apoplast.

The third model (passive diffusion) is supported by molecular dynamics simulations (*in silico*), which suggest that uncharged monolignols diffuse faster through a *Zea mays* root membrane than, e.g., glucosylated derivatives (Vermaas et al., 2019). Bulky uncharged lignin compounds, such as tricin, passed membranes even faster than uncharged lignin monomers (Vermaas et al., 2019), whereas the transport rates for both monolignols and tricin were calculated to be sufficient for depositing normal amounts of lignin. This suggests that charged lignin precursors require active transport, whereas uncharged precursors can diffuse passively through the plasma membrane. The latter mechanism might complement the active transport of uncharged monolignols by ABC transporters and serve as an alternative secretion path, for example, in case cells are energy-deficient. Furthermore, it is possible that active precursor transport is required as long as lignin polymer-producing laccases and peroxidases are scarcely present/active in the apoplast, whereas passive diffusion becomes sufficient as soon as precursors are rapidly incorporated into the lignin polymer (Perkins et al., 2019).

The fourth transport model (transport *via* channels) lacks support by experimental evidence. Future studies may mine membrane proteomes for candidate channels and additionally test whether any facilitated diffusion mechanisms (e.g., *via* lipid transfer proteins; LTPs) support lignin precursor transport.

Taken together, secretion of lignin precursors into the apoplast is a relatively poorly understood process and potentially involves a sophisticated network of transport mechanisms and transporters located in different cellular compartments. Different plant species and organs can exhibit different sets of transporters to traffic different lignin precursors. It is also possible that altered environmental conditions may impact the predominating transport mechanism (Moura et al., 2010). Thus, future studies might address (1) why plants require more than one secretion path and (2) how plants coordinate different transport mechanisms to produce one polymer.

### Suberin

Similarly to lignin, suberin is a structural and protective polymer in the plant apoplast. It plays a substantial role in regulating water and nutrient uptake and resisting soil-borne microbes in roots; in stems, suberin strengthens, and protects tissues (Vishwanath et al., 2015). Suberin contains some monolignols and hydroxycinnamic acids (*p*-coumaric and ferulic acid), which are ester-bonded to the predominant aliphatic domains (Franke and Schreiber, 2007; Vishwanath et al., 2015; Figure 1). The aliphatic domains consist of long-chain fatty acids (C_16–24_) and epoxy derivatives with minor primary fatty alcohols and glycerol (Franke et al., 2005; Pinto et al., 2009; Figure 1). The aromatic domains might adhere suberin to lignin and to the polysaccharide-rich cell wall fraction *via* ester bonds (Graça and Santos, 2007; Serra et al., 2010).

The export of suberin precursors (2-acylglycerol-3-phosphates and aliphatic hydroxycinnamate conjugates) (Li et al., 2007; Vishwanath et al., 2015; Figure 2) is poorly understood. Current evidence suggests that half-size ABC transporters are involved (Figure 2). These transporters differ from later diverged full-size ABC transporters (e.g., ABCG29; see above) as they require homo- or hetero-dimerization with another half-size ABC transporter to be functional (Gräfe and Schmitt, 2020). Based on the co-expression with suberin biosynthesis genes, the half-size transporters AtABCG2, 6, and 20 are likely to transport suberin precursors (Yadav et al., 2014). Knocking out all of them (triple mutant) resulted in an appreciably increased permeability of seed coats and roots. This observation was attributed to local disturbances in the suberin macromolecular structure. Another half-size ABC transporter, AtABCG11, might also be involved in exporting suberin precursors because roots in silenced *Arabidopsis thaliana* lines contained reduced amounts of aliphatic suberin components (Panikashvili et al., 2011). Recently, Shanmugarajah et al. (2019) suggested that AtABCG1 contributes to suberin precursor export because it traffics C_24_ α,ω-diacids and C_26_ fatty acids and alcohols–suberin building blocks–in roots and *in vitro*. Taken together, a set of half-size ABC transporters has been identified to export suberin precursors in roots, suggesting a functional redundancy. This redundancy needs to be verified by systematically testing the substrate specificities of these ABC transporters for both aliphatic and aromatic suberin precursors.

Lipid transfer proteins might be involved in suberin precursor secretion too (Edqvist et al., 2018; Figure 2). LTPs are small soluble proteins (<10 kDa) that contain a hydrophobic pocket to bind lipid monomers and thus potentially accept aliphatic suberin precursors too. LTPs can diffuse to the site of deposition in the apoplast due to their hydrophilic surface (Edqvist et al., 2018; Salminen et al., 2018). Lee and Suh (2018) suggested that a group of non-specific LTPs (e.g., AtLTPG-15), which bind to the plasma membrane *via* a glycosylphosphatidylinositol-anchor, might govern suberin monomer export in seeds because the suberin aliphatic components are significantly decreased in knockout mutants. AtLTPI-4, a non-specific LTP that diffuses freely in the apoplast (Salminen et al., 2018), was found to be upregulated in suberin-induced *A. thaliana* inflorescence stems (Deeken et al., 2016), whereas the corresponding knockout mutants showed a decreased suberin deposition. *In vitro*, AtLTPI-4 transported C_24–28_ fatty acids, which mimic suberin precursors (Deeken et al., 2016). However, the mode of action of LTPs for suberin precursor transport remains unknown (Figure 2). It was suggested that LTPs take over suberin precursors that have been exported by ABC transporters and then diffuse to the site of deposition (Edqvist et al., 2018). In case precursors were docked by membrane-anchored LTPs, the cargos are further transferred to diffusing LTPs (Figure 2). In the future, the actual role of LTPs at the molecular level could be assessed by super-resolution microscopy, which would allow for tracking LTPs’ interactions with ABC transporters and various suberin precursors in live cells. Click-chemistry or other biorthogonal labeling strategies could be employed to track suberin precursors *in planta*.

Surprisingly, even though suberin aliphatic components are known to be synthesized in the ER, there is only one report on the role of secretory vesicles in exporting suberin precursors (Oparka and Gates, 1982). Passive diffusion is another potential secretion mechanism as the aliphatic hydroxycinnamate can be hydrophobic enough to pass through the plasma membrane. However, this has not been investigated yet.

In summary, half-size ABC transporters are considered the dominant mechanism for exporting suberin precursors and might receive assistance from LTPs. It is unknown why only half-sized ABCG transporters were found for suberin export. However, it is possible that full-size suberin exporters have been overlooked so far. Thus, finding full-size ABC transporters that transport suberin and half-size transporters for lignin would be an exciting new perspective in the field of precursor export.

### Cutin

Cutin forms a polyester layer like suberin but contains less phenolics and shorter hydroxy fatty acids [e.g., 10,16-dihydroxyhexadecanoic acid (C_16_); Fich et al., 2016; Figure 1]. Cutin biosynthesis may have emerged during terrestrialization (∼480 million years ago; Morris et al., 2018), allowing streptophytes to restrict water loss (Philippe et al., 2020). Currently, knowledge on the cutin precursor export is scarce, whereas its composition, apoplastic metabolism, and function catch more attention (Yeats et al., 2012; Berhin et al., 2019; Diarte et al., 2019; Bourgault et al., 2020; Natarajan et al., 2020).

As found for lignin and suberin, cutin precursors are secreted *via* ABC transporters (Figure 2). Silencing the half-size ABC transporter AtABCG11 – a potential transporter for suberin and lignin precursors too (Takeuchi et al., 2018) – caused fusions of vegetative organs, which is a typical cutin-defective phenotype (Panikashvili et al., 2010). Moreover, the content of two cutin C_18_ dicarboxylic fatty acids in the cuticle was significantly reduced in siliques of mutant plants. Another half-size ABC transporter, AtABCG13, was found to export cutin precursors, the knockout lines displayed strong phenotypes in flowers (e.g., inter-organ fusion), and the content of most cutin monomers decreased to ∼50% of the wild-type (Panikashvili et al., 2011). Recently, a full-size transporter, AtABCG32, was reported to contribute to cuticle formation because knockout mutants exhibit slightly less cutin deposition in leaves (Fabre et al., 2016). Authors emphasized that the exact function of ABCG32 remains vague because there is no evidence for it to be indeed required for exporting 2-monoacylglycerols, the only verified cutin precursor type (Yeats et al., 2012; San Segundo et al., 2019). The same applies to AtABCG11 and 13.

ATP-binding cassette transporters might be responsible for transporting cutin precursors from the cytoplasm to the apoplast. However, unlike lignin and suberin, the hydrophobic cutin precursors have to pass through the hydrophilic cell wall to become part of the outermost cuticle layer. LTPs and cutinsomes (colloids with a cutin-precursor core and a hydrophilic shell made of cell wall components) could be responsible for this *trans-*cell wall transport (Figure 2). Experimental evidence for a role of LTPs is missing; however, high expression levels of the GPI-linked LTP MiLTPG2 were found to correlate with the cutin accumulation peak in mangos (Tafolla-Arellano et al., 2020). Cutinsomes might diffuse to the cutin deposition sites with the force of transpiration, which generates a water flow toward the epidermal cell surface that might be strong enough to carry cutinsomes with it (Domínguez et al., 2010; Segado et al., 2020).

### Cutan

Cutan is a polyether composed of C_4–33_ α,ω-diacids, alkanes, and alkenes (Villena et al., 1999; Boom et al., 2005; Guzmán-Delgado et al., 2016; Figure 1). Cutan was first characterized in leaves of *Agave americana* and *Clivia miniata* decades ago (Nip et al., 1986) and is considered to be restricted to drought-adapted plants (Boom et al., 2005; Guzmán-Delgado et al., 2016). Even though the correlation between cutan occurrence and drought-adaptation is strong, it is not clear whether cutan is indeed an adaption to low water availability (Boom et al., 2005). To further study cutan’s role for desiccation resistance, cutan-deficient plants would be required. However, information on cutan biosynthesis and transportation is missing. As a potentially considerable carbon sink in some plants, cutan deserves more attention, and a better understanding of its formation might bring new insight into plant’s strategies to cope with low water availability.

## Discussion

So far, only transporters belonging to the ABCG group have been verified as part of the active transport mechanism for lignin, suberin, and cutin precursors. The apparent dominance of these transporters might be due to their membrane topology, which allows for trafficking hydrophobic compounds. To test this hypothesis, crystallization of ABCGs with ligands can be used to qualify the interacting mode. A prominent bottleneck in the field is the lack of sensitive techniques that allow for tracking the movement of precursors in living cells. For example, there is no experimental evidence for the hypothesized passive diffusion of lignin precursors through membranes. To provide direct evidence on how cells transport various lipid-like molecules, trackable precursors (e.g., *via* click-chemistry) would be an excellent toolset for high-resolution live-cell imaging.

Overall, elucidating transmembrane export mechanism for hydrophobic molecules is an exciting field and deserves more attention. Gained knowledge will help to assess how plants react to climate change scenarios, as these polymers are fundamental for regulating the plant water household. Future studies may investigate whether plants can adjust their biopolymer compositions through switching between certain precursor transport mechanisms. This would open new opportunities for engineering plants as it would allow for regulating, e.g., the lignin content by modifying transporters rather than amending the lignin biosynthesis machinery, which could interfere with the cellular metabolism. Biomedically, new insights into the intriguing mechanism of cutin transport through the cell wall could inspire new drug delivery systems, such as nanocarriers that do not only pass through both hydrophilic and hydrophobic barriers but also target their destination with high efficiency.

## Author Contributions

AX drafted the manuscript and Figures 1, 2. KH edited them. Both authors approved the final manuscript.

## Conflict of Interest

The authors declare that the research was conducted in the absence of any commercial or financial relationships that could be construed as a potential conflict of interest.
